# Dopant-tuned stabilization of intermediates promotes electrosynthesis of valuable C3 products

**DOI:** 10.1038/s41467-019-12788-0

**Published:** 2019-10-22

**Authors:** Tao-Tao Zhuang, Dae-Hyun Nam, Ziyun Wang, Hui-Hui Li, Christine M. Gabardo, Yi Li, Zhi-Qin Liang, Jun Li, Xiao-Jing Liu, Bin Chen, Wan Ru Leow, Rui Wu, Xue Wang, Fengwang Li, Yanwei Lum, Joshua Wicks, Colin P. O’Brien, Tao Peng, Alexander H. Ip, Tsun-Kong Sham, Shu-Hong Yu, David Sinton, Edward H. Sargent

**Affiliations:** 1 Department of Electrical and Computer Engineering, University of Toronto, 35 St George Street, Toronto, ON M5S 1A4 Canada; 2 Division of Nanomaterials & Chemistry, Hefei National Laboratory for Physical Sciences at Microscale, Collaborative Innovation Center of Suzhou Nano Science and Technology, CAS Center for Excellence in Nanoscience, Department of Chemistry, University of Science and Technology of China, Hefei, Anhui 230026 P. R. China; 3 Department of Mechanical and Industrial Engineering, University of Toronto, 5 King’s College Road, Toronto, ON M5S 3G8 Canada; 4 Department of Chemistry, University of Western Ontario, 1151 Richmond Street, London, ON N6A 5B7 Canada

**Keywords:** Electrocatalysis, Electrocatalysis, Carbon capture and storage, Electrocatalysis

## Abstract

The upgrading of CO_2_/CO feedstocks to higher-value chemicals via energy-efficient electrochemical processes enables carbon utilization and renewable energy storage. Substantial progress has been made to improve performance at the cathodic side; whereas less progress has been made on improving anodic electro-oxidation reactions to generate value. Here we report the efficient electroproduction of value-added multi-carbon dimethyl carbonate (DMC) from CO and methanol via oxidative carbonylation. We find that, compared to pure palladium controls, boron-doped palladium (Pd-B) tunes the binding strength of intermediates along this reaction pathway and favors DMC formation. We implement this doping strategy and report the selective electrosynthesis of DMC experimentally. We achieve a DMC Faradaic efficiency of 83 ± 5%, fully a 3x increase in performance compared to the corresponding pure Pd electrocatalyst.

## Abstract (web summary)

The electro-oxidative synthesis of valued chemicals offers to enhance the overall efficiency and economic viability of renewable electrosynthesis systems. Here, the authors use dopant-tuned catalysts to promote the electrosynthesis of dimethyl carbonate from CO and methanol via oxidative carbonylation.

## Introduction

The cathodic carbon dioxide (CO_2_) reduction reaction has seen rapid progress of late, including in the production of CO, methane, formic acid, ethylene, ethanol, and propanol^1–8^. At present, most electrochemical anodic side reactions have utilized the oxygen evolution reaction (OER).

Anodic reactions offer, in principle, valuable upgrades of waste products and lower-value commodity chemicals; yet have seen less exploration in electrocatalysis^9–12^. In anodic chemical upgrade reactions, a particularly important challenge is to achieve selective electro-oxidation to the desired valuable product, instead of overoxidizing the inputs all the way to CO_2_.

Industrial effluent streams and steel flue gas contain CO, a high-energy feedstock that nonetheless commands a low market value^13–15^. The impressive progress of CO_2_-to-CO using electrocatalysis further motivates exploring ways to upgrade CO produced from CO_2_ to more valuable products.

Here we explore coupling methanol and CO via electrochemical oxidative carbonylation to dimethyl carbonate^16–18^ (DMC, Eq. (1)). This enables the production of a valuable C3 compound—one already industrially important as a fuel additive, as a polar solvent, and as an environmentally sustainable intermediate for the upgrade of several promising renewables^19,20^. The global market for DMC will exceed $500M USD by 2025^21^ and our technoeconomic assessment (TEA, Supplementary Figs. 1, 2, and Table 1) indicates a production cost of US$1200/ton for DMC from total chemical + renewable electricity inputs to be ~US$600/ton.

To catalyze the electrosynthetic pathway

1 $$2{\mathrm{{CH}}}_{\mathrm{3}}{\mathrm{OH}} + {\mathrm{{CO}}}-2{\mathrm{{e}}}^ - \to \left( {\mathrm{{{CH}}}_{3}{\mathrm{O}}} \right)_2{\mathrm{{CO}}} + 2{\mathrm{{H}}}^ +$$

palladium (Pd)-based electrodes achieve methanol carbonylation with CO^22,23^, but suffer from the formation of by-products, such as dimethyl oxalate, lowering the DMC selectivity.

We first investigated the reaction steps with the goal of further understanding mechanism; and then used these insights to engineer catalysts to increase selectivity to DMC production. We utilized density functional theory (DFT) to explore what controls the binding strength of intermediates on the catalyst along the methanol-CO to DMC pathway. Our findings motivate us to attempt the doping of Pd to tune intermediate binding energies to favor DMC formation, a strategy we implemented experimentally, allowing us to achieve high-faradaic-efficiency conversion to DMC. This work suggests further potential in interstitial doping to promote oxidation-based carbon upgrade reactions using renewable feedstocks.

## Results

### Computational studies

**Fig. 1:**
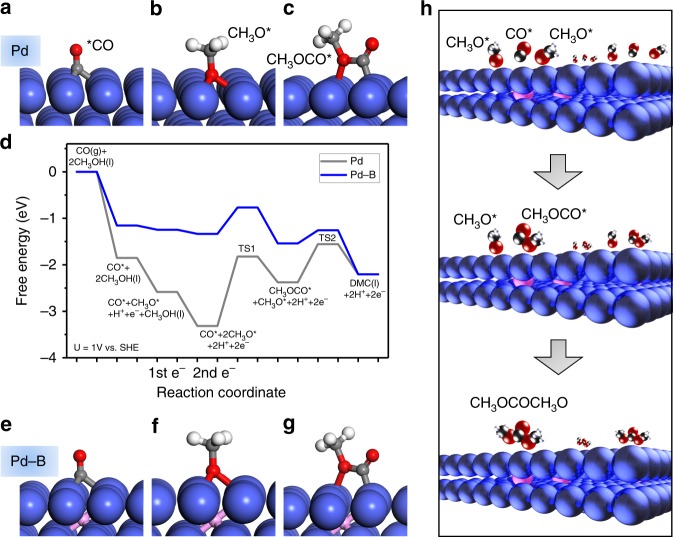
DFT calculations on DMC formation. Geometries of intermediates in DMC formation reactions: **a** CO, **b** CH_3_O, **c** CH_3_OCO on pure Pd; **e** CO, **f** CH_3_O, **g** CH_3_OCO on boron-doped Pd (Pd–B). **d** Free energy profiles of DMC formation from CO and CH_3_OH on pure Pd (gray) and Pd–B (blue) at *U* = 1 V vs. SHE. **h** Reaction scheme for electroproduction of DMC from methanol and CO on the Pd–B electrode. DMC/CH_3_OCOCH_3_O: dimethyl carbonate. Red, gray, white, pink, and blue balls represent oxygen, carbon, hydrogen, boron, and palladium, respectively

We first investigated, using computational studies, the reaction of methanol-CO to DMC on Pd(111). The DMC formation reaction begins with CO adsorption to form CO* (Fig. 1a) and the dissociation of CH_3_OH into CH_3_O* (Fig. 1b). These intermediates undergo coupling to form CH_3_OCO* (Fig. 1c). DMC is then generated through the formation of a C–O bond between CH_3_OCO* and another CH_3_O*. The dissociation of CH_3_OH is the only step among these that involves electron transfer (implicated in proton coupled electron transfer)^24^. We then evaluated the effect of applied potential on the reaction using the computational hydrogen electrode of Nørskov and co-workers^25^ and applied 1 V vs. SHE toward DMC electrocatalytic formation^16^.

The energy profile (Fig. 1d and Supplementary Table 2) indicates strong binding of the adsorbed CO* and the dissociated CH_3_O* on pure Pd. It is so strong as to render further coupling of CO* and CH_3_O* unfavorable. The barriers associated with the C–O bond formation steps including OC–OCH_3_ (TS1) and CH_3_O–C(O)OCH_3_ (TS2) are 1.49 and 0.82 eV (Fig. 1d and Supplementary Fig. 4), respectively, indicating that coupling is also unfavorable kinetically. Clearly, tuning the binding strength of the catalyst has the potential to improve DMC formation.

We investigated boron doping of Pd in the context of the anodic oxidative carbonylation reaction (Fig. 1e–g, Supplementary Fig. 3 and Supplementary Tables 3–5). The results (Fig. 1d) reveal that boron doping controls the adsorption of both CO* and CH_3_O*, rendering the energies of intermediates along the reaction process downhill toward CH_3_OCO* formation. Furthermore, the barriers are 0.57 eV for TS1 and 0.28 eV for TS2 on boron-doped palladium (Pd–B), which are significantly lower compared to those on Pd (Fig. 1d), thus indicating increased selectivity to DMC electrosynthesis on Pd–B (Fig. 1h).

### Catalyst synthesis and characterization

**Fig. 2:**
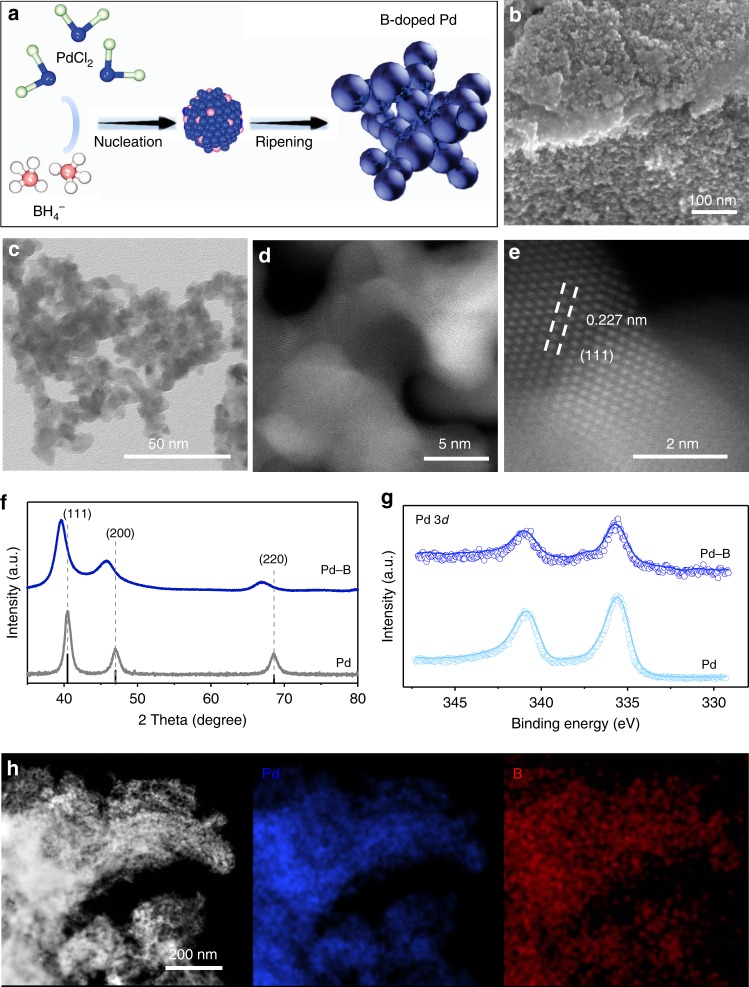
Catalyst synthesis and structure characterization. **a** The fabrication schematic illustration of Pd–B catalysts. **b** SEM, **c** TEM, **d** HAADF, and **e** HAADF-STEM images, showing the morphology and size of Pd–B nanomaterials. **f** Powder XRD spectra of Pd–B and Pd samples show a peak shift, demonstrating the boron penetration into the Pd lattice. Black line corresponding to JCPDS No. 05-0681. **g** XPS spectra for Pd 3*d* regions. **h** EELS, showing the homogeneous distribution of Pd and B elements in the Pd–B sample. The scale bar in **h** is 200 nm

Experimentally we prepare Pd–B interstitial nanoalloys (Fig. 2a, details in the “Methods” section). We used scanning electron microscopy (SEM), transmission electron microscopy (TEM), and high-angle annular dark-field scanning transmission electron microscopy (HAADF-STEM) (Fig. 2b–d) to examine the morphology and size of the resultant Pd–B. We obtained a dendritic Pd–B morphology comprised of nanoparticles ranging in diameter from 5 to 10 nm.

From TEM, the Pd–B lattice spacing is 0.227 nm (Fig. 2e), larger than that of pure Pd (0.222 nm, Supplementary Fig. 5), consistent with lattice expansion when B atoms penetrate into the Pd lattice. Powder X-ray diffraction (PXRD) confirms the same trend: the diffraction peak of Pd–B shifts to a lower 2*θ* value compared to that of the Pd control (Fig. 2f)^26,27^. X-ray photoelectron spectroscopy (XPS) confirms the change in the electronic structure of Pd via B-doping as seen in the slight shift of Pd 3*d*-binding energy (Fig. 2g), while electron energy loss spectroscopy (EELS) mapping reveals that Pd and B are uniformly distributed in the Pd–B sample (Fig. 2h).

We tuned the B concentration inside the Pd nanocrystals by varying the precursor ratio between Pd and B (see the “Methods” section). The sample retained a similar size and shape as we increased B content, and lattice fringes expanded and the diffraction peak shifted further (Supplementary Figs. 6 and 7).

**Fig. 3:**
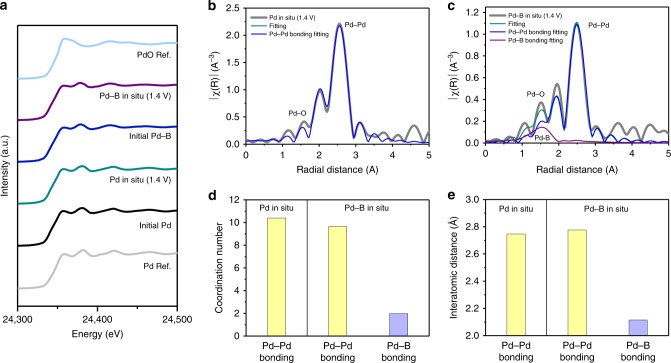
*Operando* XAS of catalysts during DMC electroproduction. **a** Pd K-edge XAS for investigating the Pd and Pd–B catalysts at the potential of 1.4 V vs. Ag/AgCl. **b**, **c** *Operando* EXAFS of (**b**) Pd and (**c**) Pd–B to investigate the local atomic structure around the Pd atom with Pd–Pd and Pd–B fitting. **d**, **e** EXAFS fitting induced (**d**) coordination number and (**e**) interatomic distance comparison between Pd and Pd–B

We then investigated, using *operando* X-ray absorption spectroscopy^28^ (XAS), the stability of Pd–B during DMC electroproduction, investigating both pure Pd and Pd–B electrocatalysts (Fig. 3). We probed the local environment in the vicinity of the Pd atoms by tracking changes in X-ray absorption near edge structure (XANES) and extended X-ray absorption fine structure (EXAFS) spectra. These were measured during anodic reaction under a potential of 1.4 V vs. Ag/AgCl in 0.1 M NaClO_4_/methanol electrolyte with continuous CO gas flowing.

Pd K-edge XANES of pure Pd and Pd–B electrocatalysts revealed no evident change in Pd valence state during the reaction (Fig. 3a). We found that the electrode composition was stable across the reaction time (XPS, Supplementary Fig. 8). When we compared the average oxidation state of Pd at 1.4 V vs. Ag/AgCl, the XANES linear combination fitting of Pd–B resulted in +0.68 as an average oxidation state, which is higher than the average oxidation state of pure Pd, +0.59 (Supplementary Table 6). Both pure Pd and Pd–B showed lower oxidation states than the +2 of PdO. We attribute the higher Pd oxidation state of Pd–B to bonding between Pd and B.

Using EXAFS to acquire information on atomic bonding near the Pd atom (Fig. 3b and c), we found that Pd–B has a lower Pd–Pd coordination number (Pd: 10.4, Pd–B: 9.7) and longer Pd–Pd interatomic distance (Pd: 2.747 Å, Pd–B: 2.776 Å) compared to that of pure Pd (Fig. 3d, e) during electrocatalytic DMC production when B is present (Supplementary Fig. 9). Pd–B fitting of EXAFS (Pd–B coordination number: 1.98, Pd–B interatomic distance: 2.115 Å) indicates the same trend. We conclude that the interstitial B doping in the Pd lattice is stable across reaction times studied herein.

### Electrochemical oxidation reaction investigations

**Fig. 4:**
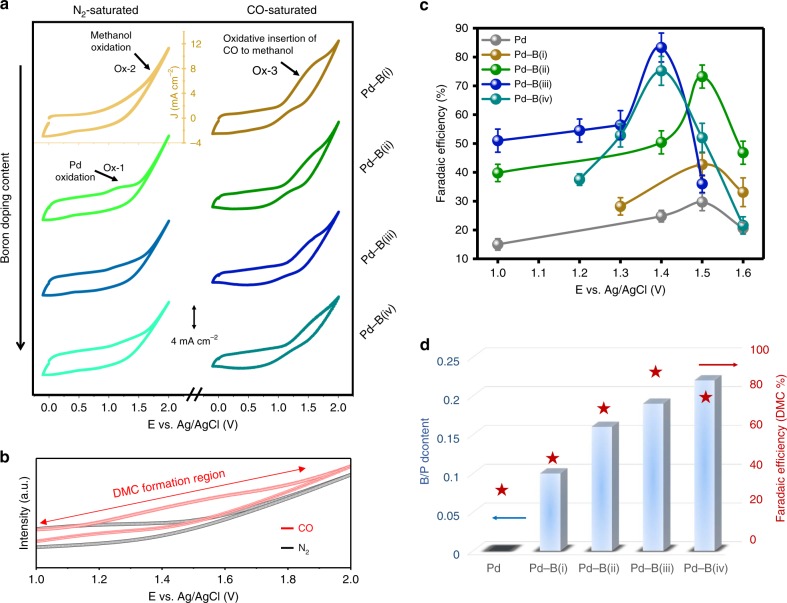
Effects of boron doping in palladium on the DMC electroproduction. **a** Cyclic voltammograms on the Pd–B electrodes, 0.1 M NaClO_4_/methanol electrolytes; scan rate 50 mV S^−1^. Left: N_2_-saturated; right: CO-saturated. **b** amplified CV, highlighting DMC formation region. **c** DMC Faradaic efficiencies on the catalysts under different applied potentials show the Pd–B (iii) sample has the best selectivity. **d** The effect of B doping content in Pd on DMC selectivity. Error bars correspond to the standard deviation of three or more measurements

We deposited catalysts onto carbon paper via spray coating (see the “Methods” section) and characterized the electrochemical CO–methanol oxidative carbonylation activity and selectivity toward DMC using a three-electrode H-cell configuration. We first measured cyclic voltammograms (CV) of the anodes to study electrocatalytic carbonylation. In nitrogen-purged electrolytes (0.1 M NaClO_4_/methanol), we observed a broad oxidation peak (Ox-1) for all Pd–B samples at ca. 1.2 V that we ascribe to the electrochemical oxidation of Pd^0^ to Pd^2+^ with methanol (Eq. (2), Fig. 4a left, and Supplementary Fig. 10)^23^. A steep increase in current was seen at potentials higher than 1.5 V (Ox-2) owing to direct methanol oxidation (Eqs. (3) and (4))^22^. Upon bubbling and saturation of the solution with CO, the oxidative insertion of CO into methanol occurred (broad oxidation peak at ca. 1.5 V, Ox-3)^29^. This implied DMC formation (Fig. 4b), and the product was further evaluated by gas chromatography with flame-ionization detection and gas chromatography with mass spectrometry (GC-FID and GC-MS, Supplementary Figs. 11 and 12), respectively. CO–methanol oxidative carbonylation suppressed the Pd electrode self-oxidation evidenced by XAS data in Fig. 3a, and it also shifted the large current of direct methanol oxidation to more positive potentials (>1.8 V).

2 $${\mathrm{{Pd}}}^0 + 2{\mathrm{CH}}_{3}{\mathrm{OH}} \to {\mathrm{{Pd}}}^{2+}\left( {{\mathrm{CH}}_{3}{\mathrm{O}}^ - } \right)_2 \, + \, 2{\mathrm{{H}}}^ +$$

3 $$3{\mathrm{CH}}_{3}{\mathrm{OH}}-2{\mathrm{{e}}}^ - \to \left( {{\mathrm{CH}}_{3}{\mathrm{O}}} \right)_2{\mathrm{{CH}}}_2 + 2{\mathrm{{H}}}^ + + {\mathrm{{H}}}_{2}{\mathrm{O}}$$

4 $$2{\mathrm{CH}}_{3}{\mathrm{OH}}-4{\mathrm{{e}}}^ - \to {\mathrm{{HC}}}\left( {\mathrm{O}} \right){\mathrm{{OCH}}}_3 + 4{\mathrm{{H}}}^ +$$

We then evaluated the CO–methanol oxidation performance in the potential range of 1.0–1.6 V versus Ag/AgCl (CO–methanol coupling region) in 0.1 M NaClO_4_/methanol. In this way we would investigate further the effect of the B dopant on DMC selectivity. Compared to pure Pd, all Pd–B samples showed higher DMC selectivity (Fig. 4c and Supplementary Table 8). At 1.4 V vs. Ag/AgCl, we achieved Faradaic efficiency of 83 ± 5% for DMC using the Pd–B(iii) catalyst.

This DMC production represents a three-fold improvement in selectivity compared to unmodified Pd (Supplementary Table 8 and 9). We analyzed the B dopant amount through inductively coupled plasma mass spectrometry (ICP–MS, Supplementary Table 10) and report as a result the B doping present in the catalyst (Fig. 4d) and correlate this descriptor with high DMC electrosynthesis.

## Discussion

Selective electroproduction of the C3 liquid chemical DMC from lower-value CO and methanol was achieved by oxidative carbonylation with the aid of a new boron-doped palladium electrocatalyst. Boron improved DMC selectivity verified through DFT calculations, material structure analysis, and electrochemical measurements. This work offers an avenue to upgrade carbon via electro-oxidation that could be applied to electrolyzers to achieve high-value products from both the cathodic and the anodic reactions. Ultimately, tandem electrocatalytic cathode–anode systems uniting CO_2_-to-CO (cathodic reduction) with CO-to-DMC (anodic oxidation) stand to offer integrated DMC production from CO_2_.

## Methods

### DFT calculations

In this work, all DFT calculations were carried out with a periodic slab model using the Vienna ab initio simulation program (VASP)^30^ (https://www.vasp.at/). Detailed theoretical methods can be found in Supplementary Methods.

### Catalyst synthesis

Pd–B nanoparticles^31^ were prepared via the rapid chemical reduction reaction between palladium chloride (PdCl_2_, Sigma-Aldrich) and sodium borohydride (NaBH_4_, Sigma-Aldrich). PdCl_2_ (89 mg) was dissolved in 2.5 mL deionized (DI) water. The B dopant concentration was controlled by dissolving NaBH_4_ in 12.5 mL DI water (62.5, 250, 500, 1000 mg). NaBH_4_ solution was placed in PdCl_2_ solution. After the reaction between PdCl_2_ and NaBH_4_, the Pd–B nanoparticles were washed using DI water. After centrifuging, Pd–B nanoparticles were dried in a vacuum oven overnight. Pure Pd nanoparticles were prepared using hydrazine (N_2_H_4_, Sigma-Aldrich) as a reducing agent for PdCl_2_ instead of NaBH_4_.

### Working electrode preparation and oxidation measurements

To prepare electrodes, we deposited 10 mg of catalyst mixed with 20 μl of 5 wt% Nafion in 1 mL methanol on a carbon gas-diffusion layer with loading ca. 1 mg cm^−2^ using an air-brush spray coater.

Electrocatalytic measurements were carried out in a three-electrode system using an electrochemical station (PGSTAT204, Metrohm Autolab). Electrolysis was carried out in a two-compartment electrochemical H-cell with a proton exchange membrane (Nafion 117) as the separator. All potentials were measured against a Ag/AgCl reference electrode (3 M KCl, BASi) and a platinum counter electrode. In the H-cell, the electrolyte was 0.1 M NaClO_4_/methanol saturated with CO, which was delivered into the anodic compartment at a rate of 30.00 standard cubic centimeters per minute (s.c.c.m.).

### Dimethyl carbonate analysis

The reacted solution was collected and quantitative analysis. The dimethyl carbonate (DMC) product was carried out using a capillary gas chromatograph (PerkinElmer Clarus 580 and Clarus SQ 8C with FID and MS detectors, respectively) with Stabilwax column (fused silica, Restek). The Faradaic efficiency (FE) of DMC was calculated from the total amount of charge *Q* (in units of C) passed through the sample and the total amount of the DMC produced *n* (in moles). *Q* = *I* × *t*, where *I* (in amperes) is the oxidation current at a specific applied potential and *t* is the time (in seconds) for the constant oxidation current.

The FE of the DMC is calculated as follows:

5 $${\mathrm{FE}}_{{\mathrm{DMC}}} = 2 \times F \times \frac{{n_{{\mathrm{DMC}}}}}{Q} \times {\mathrm{100\% }} = 2 \times F \times \frac{{n_{{\mathrm{DMC}}}}}{{{(I} \times {t)}}} \times {\mathrm{100\% }}$$

## Supplementary information

Supplementary Information

## Data Availability

The data that support the findings of this study are available from the corresponding author on reasonable request.

## References

[1] Raciti D, Livi KJ, Wang C (2015). Highly dense Cu nanowires for low-overpotential CO_2_ reduction. Nano Lett..

[2] Luc W (2017). Ag–Sn bimetallic catalyst with a core–shell structure for CO_2_ reduction. J. Am. Chem. Soc..

[3] Weekes DM, Salvatore DA, Reyes A, Huang A, Berlinguette CP (2018). Electrolytic CO_2_ reduction in a flow cell. Acc. Chem. Res..

[4] Nielsen DU, Hu XM, Daasbjerg K, Skrydstrup T (2018). Chemically and electrochemically catalysed conversion of CO_2_ to CO with follow-up utilization to value-added chemicals. Nat. Catal..

[5] Dinh CT (2018). CO_2_ electroreduction to ethylene via hydroxide-mediated copper catalysis at an abrupt interface. Science.

[6] Li YC (2019). Binding site diversity promotes CO_2_ electroreduction to ethanol. J. Am. Chem. Soc..

[7] Zhuang TT (2018). Copper nanocavities confine intermediates for efficient electrosynthesis of C3 alcohol fuels from carbon monoxide. Nat. Catal..

[8] Liang ZQ (2018). Copper-on-nitride enhances the stable electrosynthesis of multi-carbon products from CO_2_. Nat. Commun..

[9] Zhang B (2016). Homogeneously dispersed multimetal oxygen-evolving catalysts. Science.

[10] Li H-H (2017). Highly crystalline PtCu nanotubes with three dimensional molecular accessible and restructured surface for efficient catalysis. Energy Environ. Sci..

[11] Ma S-Y (2017). Synthesis of low Pt-based quaternary PtPdRuTe nanotubes with optimized incorporation of Pd for enhanced electrocatalytic activity. J. Am. Chem. Soc..

[12] Zheng X (2018). Theory-driven design of high-valence metal sites for water oxidation confirmed using in situ soft X-ray absorption. Nat. Chem..

[13] Verma S, Lu X, Ma S, Masel RI, Kenis PJ (2016). The effect of electrolyte composition on the electroreduction of CO_2_ to CO on Ag based gas diffusion electrodes. Phys. Chem. Chem. Phys..

[14] Jhong HRM (2017). A nitrogen‐doped carbon catalyst for electrochemical CO_2_ conversion to CO with high selectivity and current density. ChemSusChem.

[15] Gabardo CM (2018). Combined high alkalinity and pressurization enable efficient CO_2_ electroreduction to CO. Energy Environ. Sci..

[16] Figueiredo MC, Trieu V, Eiden S, Koper MT (2017). Spectro-electrochemical examination of the formation of dimethyl carbonate from CO and methanol at different electrode materials. J. Am. Chem. Soc..

[17] Davies BJ (2018). Electrochemically generated copper carbonyl for selective dimethyl carbonate synthesis. ACS Catal..

[18] Tan H-Z (2018). Review on the synthesis of dimethyl carbonate. Catal. Today.

[19] Tundo P, Selva M (2002). The chemistry of dimethyl carbonate. Acc. Chem. Res..

[20] Fiorani G, Perosa A, Selva M (2018). Dimethyl carbonate: a versatile reagent for a sustainable valorization of renewables. Green Chem..

[21] Outcalt SL, Laesecke A (2019). Compressed-liquid densities of the binary mixture dimethyl carbonate + heptane at three compositions. J. Mol. Liq..

[22] Yamanaka I, Funakawa A, Otsuka K (2002). Selective carbonylation of methanol to dimethyl carbonate by gas–liquid–solid-phase boundary electrolysis. Chem. Lett..

[23] Yamanaka I, Funakawa A, Otsuka K (2004). Electrocatalytic synthesis of DMC over the Pd/VGCF membrane anode by gas–liquid–solid phase-boundary electrolysis. J. Catal..

[24] Huynh MHV, Meyer TJ (2007). Proton-coupled electron transfer. Chem. Rev..

[25] Nørskov JK (2004). Origin of the overpotential for oxygen reduction at a fuel-cell cathode. J. Phys. Chem. B.

[26] Chan CWA (2014). Interstitial modification of palladium nanoparticles with boron atoms as a green catalyst for selective hydrogenation. Nat. Commun..

[27] Vo Doan, T. T. et al. Frontispiece: theoretical modelling and facile synthesis of a highly active boron‐doped palladium catalyst for the oxygen reduction reaction. *Angew. Chem. Int. Ed*. **55**, 6842–6847 (2016). 10.1002/anie.201601727 27086729

[28] Li J (2018). Copper adparticle enabled selective electrosynthesis of n-propanol. Nat. Commun..

[29] Funakawa A, Yamanaka I, Takenaka S, Otsuka K (2004). Selectivity control of carbonylation of methanol to dimethyl oxalate and dimethyl carbonate over gold anode by electrochemical potential. J. Am. Chem. Soc..

[30] Kresse G, Furthmüller J (1996). Efficient iterative schemes for ab initio total-energy calculations using a plane-wave basis set. Phys. Rev. B.

[31] Chen A, Ostrom C (2015). Palladium-based nanomaterials: synthesis and electrochemical applications. Chem. Rev..

