# Supplementary material for: Dopant-tuned stabilization of intermediates promotes electrosynthesis of valuable C3 products
Source: Nat Commun. 2019 Oct 22;10:4807. doi: 10.1038/s41467-019-12788-0 (PMC6805905; doi:10.1038/s41467-019-12788-0)
Supplement: Supplementary file 1 — Supplementary Information [file 41467_2019_12788_MOESM1_ESM.pdf]

Dopant-tuned stabilization of intermediates promotes electrosynthesis of valuable C3 products

Zhuang et al.

Supplementary Methods.

Techno-economic assessment. The techno-economic assessment (TEA, Supplementary Fig. 1) model was used to provide a total plant gate levelized cost of production in the units of $ per ton of dimethyl carbonate (DMC). We broadly categorized the costs into capital costs and operating costs. The capital costs are limited to the cost of the electrolyzer. The plant lifetime is assumed to be 30 years. The operating costs consist of the electricity costs, separation costs, plant operation costs, and raw material costs (here are the cost of CO and methanol).

A plant is assumed to convert 200 tons of CO[1] a day into DMC. The electrolyzer system capital cost was chosen to be $1000/kW; the electricity price was 10 cents/kWh; the separation cost was 10% of electrolyzer system cost; the plant lifetime was 10 years; and the operation cost was 10% of electricity cost via comparing the average price among the world. We assumed the capacity factor was 0.8[2], cell voltage was 2 V vs. RHE (should be achieved in the industry application), and Faradaic efficiency of DMC was 80% (the value was tuned and the corresponding TEA was shown in Supplementary Fig. 2 and Supplementary Table 1), respectively. The current price for CO and methanol are 200 $/ton and 400$/ton, respectively[3].

The production volume is:

$$\text{Production Volume (ton)} = \frac{\text{COConverted *Product Molecular Weight *Faradaic Efficiency}}{\text{CO Molecular Weight *Product Ratio*100}} \quad (1)$$

$$2CH_3OH + CO - 2e^- \rightarrow (CH_3O)_2CO + 2H^+ \quad (2)$$

$$\text{DMC production (tons/day)} = \frac{200}{28}\text{*90.08*0.8} = \text{643 tons/day} \quad (3)$$

$$\text{Methanol consume (tons/day)} = \frac{200}{28}*2*32 = 457 \text{ tons/day} \quad (4)$$

The total electricity cost is:

$$\text{Electricity cost (\$/ton)} = \frac{\text{Energy Consumed (kW)} * \text{Electricity cost} \left(\frac{\text{cents}}{\text{kWh}}\right) * \text{24hours}}{\text{Production volume (ton)} * (100\frac{\text{cents}}{\text{dollar}})}$$

$$\text{Electricity cost (\$/ton)} = \frac{\text{Energy Consumed (kW)} * 10 \left(\frac{\text{cents}}{\text{kWh}}\right) * \text{24hours}}{647 * (100\frac{\text{cents}}{\text{dollar}})} \quad (5)$$

Where the energy consumed is:

$$\text{Energy consumed (kW)} = \text{current*cell voltage} = \frac{\text{(nCO*F*electron transfer)}}{\text{Faradaic efficiency*Capacity factor*86400seconds}} =$$

$$\frac{(7142857*96485*3)}{0.8*0.8*86400\text{seconds}} = 49853.77\text{kW} \quad (6)$$

$$\text{Electricity cost (\$/ton)} = 148.76\text{\$/ton} \quad (7)$$

The capital cost (electrolyze cost) is:

$$\text{Capital cost (\$/ton)} = \frac{\text{Energy consumed*Electrolyzer cost*life time plant (days)}}{\text{Production Volume}}$$

$$\text{Capital cost (\$/ton)}=\frac{49853.77*1000*3650}{647} = 21.23\text{\$/ton} \quad (8)$$

The separation cost is:

$$\text{Separation cost (\$/ton)} = 10\%\text{*Electrolyzer cost} = 100.00\text{\$/ton} \quad (9)$$

The operation cost is:

$$\text{Operation cost (\$/ton)} = 10\%\text{*Electrocity cost} = 14.88\text{\$/ton} \quad (10)$$

Raw material cost is:

$$\text{Raw material cost (\$/ton)}$$

$$= \text{CO (cost)+mathanol (cost)}=200*\frac{28}{90.08}+400*32*\frac{2}{90.08} = 346.36\text{\$/ton} \quad (11)$$

The total plant-gate levelized cost is then described as:

$$\text{Total Cost} = \text{Capital Cost+Electricity Cost+Separation Cost+Operational Cost+Raw Material Cost}$$

$$\text{Total cost (\$/ton)} = \frac{\$21.23}{\text{ton}}+\frac{\$148.76}{\text{ton}}+\frac{\$100.00}{\text{ton}}+\frac{\$14.88}{\text{ton}}+\frac{\$346.36}{\text{ton}} = 631.23\text{\$/ton} \quad (12)$$

Theoretical methods. In this work, all the DFT calculations were carried out with a periodic slab model using the Vienna *ab initio* simulation program (VASP) [4-7]. The

generalized gradient approximation (GGA) was used with the Perdew-Burke-Ernzerhof (PBE)[8] exchange-correlation functional. The projector-augmented wave (PAW) method [9, 10] was utilized to describe the electron-ion interactions, and the cut-off energy for the plane-wave basis set was 450 eV. In order to illustrate the long-range dispersion interactions between the adsorbates and catalysts, we employed the D3 correction method by Grimme et al.[11]. Brillouin zone integration was accomplished using a 3×3×1 Monkhorst-Pack k-point mesh. All the adsorption geometries were optimized using a force-based conjugate gradient algorithm, while transition states (TSs) were located with a constrained minimization technique[12-14]. The effect of methanol solvent is considered using the implicit solvation model implemented in VASPsol[15]. The dielectric constant of methanol was set to be 33.10 at 298.15 K, 1 atm[16]. The entropy values of CO, CH_3OH, H_2, and DMC are 197.66 J/mol/K[17], 127.2 J/mol/K[17], 130.68 J/mol/K[17], and 109.66 J/mol/K[18], respectively. The entropy of surface species was ignored. For the modelling of Pd(111), the crystal structure was optimized, and Pd(111) was modelled with a periodic four-layer p(3×3) model with the 2 lower layers fixed and 2 upper layers relaxed.

To evaluate the effect of boron doping, we investigated four different boron-doped Pd(111) as shown in Supplementary Fig. 3 and Supplementary Table 2. The most stable doping site is the boron on the subsurface fcc site, and the rest doping sites are at least 1 eV unstable compared to subsurface fcc. Therefore, we used subsurface fcc as the model in our calculations.

We further carried out the Bader analysis. The Bader charge values of B and the connected three surface and subsurface Pd were listed in Supplementary Table 4.

In order to investigate the effects of higher concentration of boron doping, we calculated the adsorption energies of CO (E(CO)) and the free energy change of CH_3OH dissociation ($\Delta G(CH_3OH)$) with boron numbers from 1 to 10 in the 4×4 unit cell (Supplementary Table 5). The results suggest that with boron doping could significantly decreases both energies. These calculations suggest that the promotion effect for DMC activity is consistent with the different boron doping concentrations.

***In-situ* X-ray absorption spectroscopy (XAS).** Operando XAS was measured at 20-BM beamline (energy range: 2.7 ~ 32.7 keV) of the Advanced Photon Sources (APS) in Argonne National Laboratory (Lemont, Illinois). For the operando experiment, flow-cell type XAS reactor with the transparent backside (sealed with Kapton tape) was installed in beamline station and XAS was detected by fluorescence detector. The constant potential of 1.4 V vs. Ag/AgCl was applied to the cell with circulating the 0.1 M $NaClO_4$/methanol electrolyte (continuous CO gas flowing). The IFEFFIT was applied to analyze the XAS spectra by using Athena program. After the data calibration and normalization, EXAFS was fitted for the Pd-Pd, Pd-B coordination number and interatomic distance by Artemis program.

Characterization. Powder X-ray diffraction patterns (PXRD) were measured on a Rigaku Miniflex 600 equipped with graphite-monochromatized Cu Ka radiation. X-ray photoelectron spectroscopy (XPS) was carried out with the Thermo Scientific K-Alpha XPS system. An Al Kα source with a 400μm spot size was used for

measurements to detect photo-electrons at specific energy ranges to determine the presence of specific elements. Scanning electron microscopy (SEM) was performed with a field emission scanning electron microanalyzer (Zeiss Supra 40 and Hitachi S-5200). Transmission electron microscopy (TEM) and high-resolution transmission electron microscopy observations were performed using a Talos F200X microscope. High-angle annular dark-field imaging scanning transmission electron microscopy (HAADF-STEM) and electron energy loss spectroscopy (EELS) were carried out using an aberration-corrected STEM instrument (Titan Cubed Themis G2 300) equipped with an EELS spectrometer of Gatan Enfinium ER 977. Inductively coupled plasma mass spectrometry (ICP-MS) was carried out on Optima 7300 DV to analyze B/Pd content. Raman measurements were carried out using a Renishaw inVia Raman Microscope in a modified cell and a water immersion objective (63x) with a 785 nm laser, using a 5 s integration and averaging 10 scans per region. Ag/AgCl electrode was used as the reference electrode and a Pt wire was used as the counter electrode.

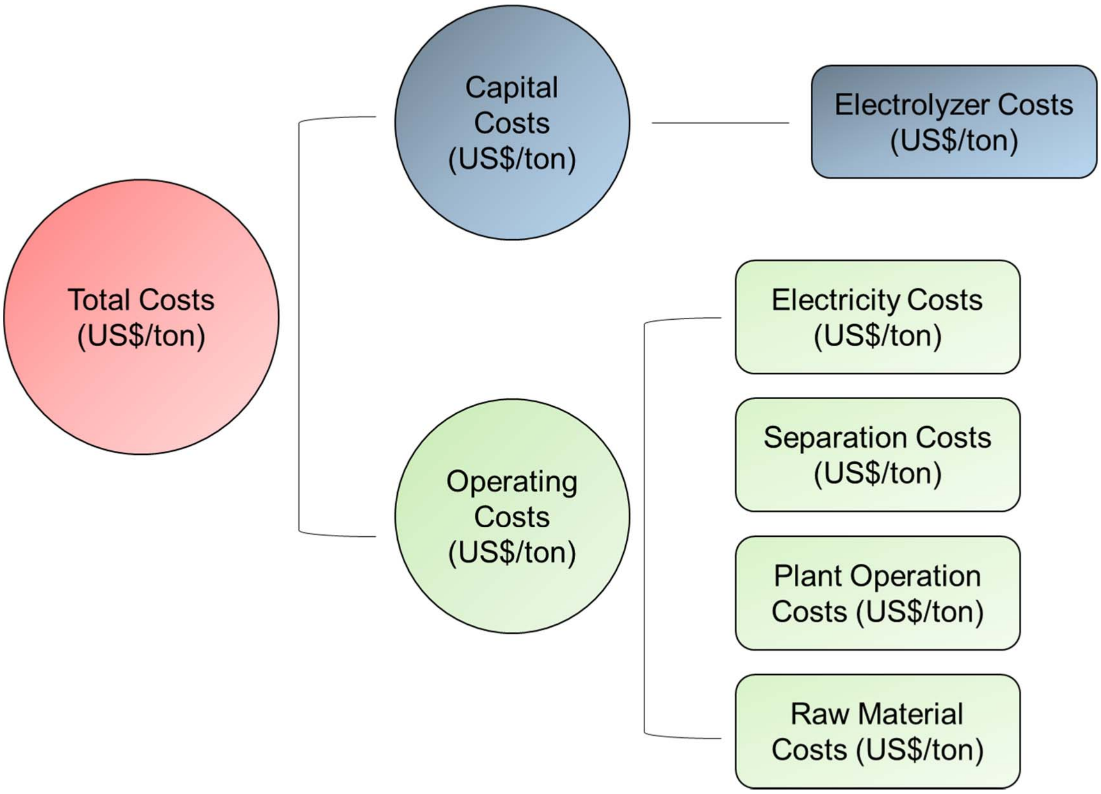

Supplementary Figure 1. The content for total costs within techno-economic assessment (TEA).

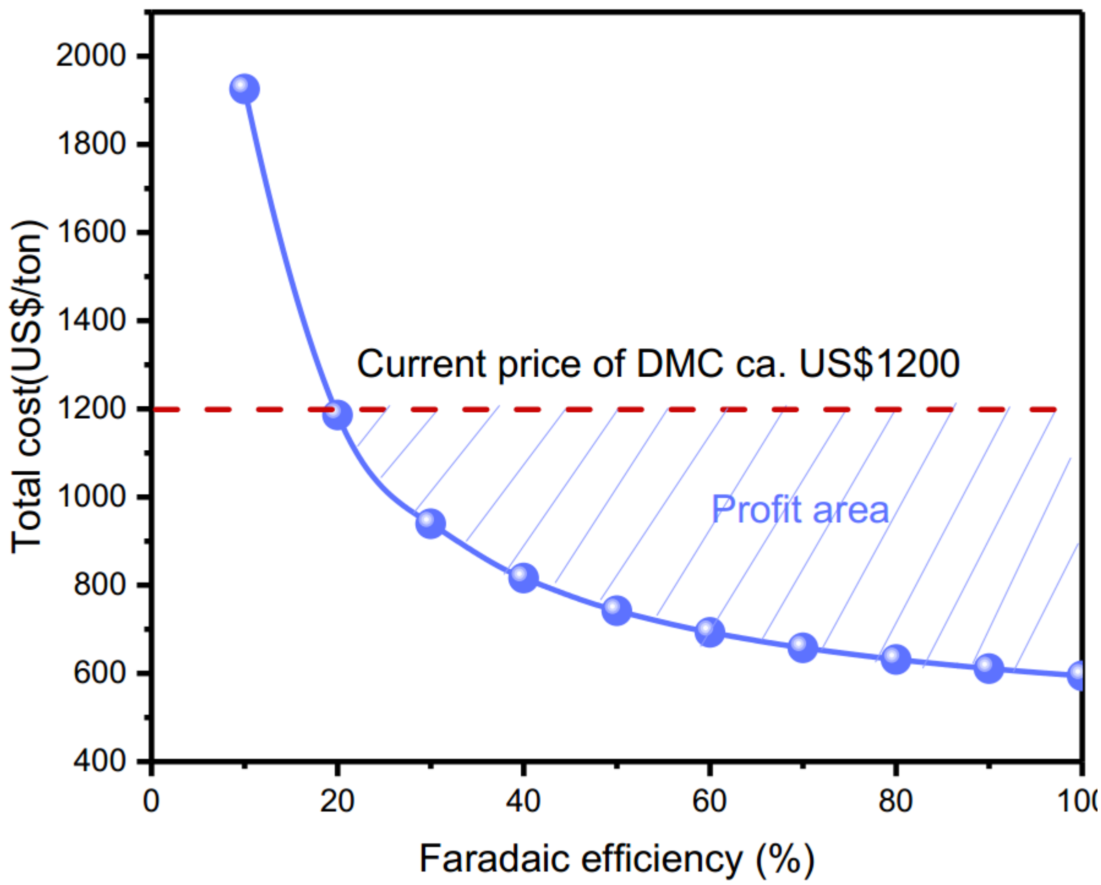

Supplementary Figure 2. The plot of production costs of electrosynthesized DMC versus DMC Faradaic efficiency. It can be profitable to electrosynthesize DMC when we achieve ca. 20% Faradaic efficiency of DMC, compared to the current market price of DMC US$1200[19].

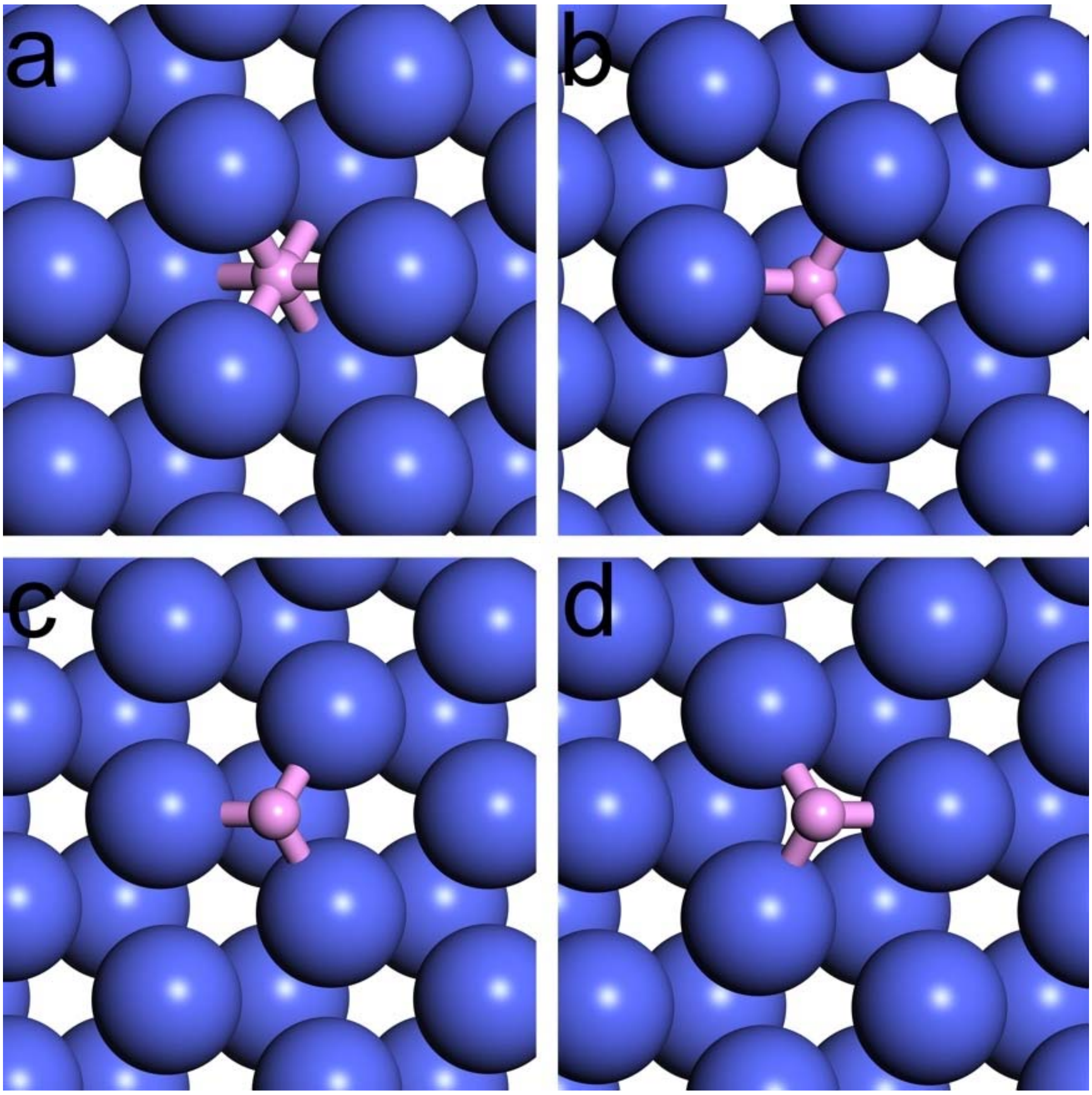

Supplementary Figure 3. Geometries of boron doped Pd with boron on (a) subsurface fcc, (b) subsurface hcp, (c) surface hcp, and (d) surface fcc. Pink and blue balls stand for boron and palladium, respectively.

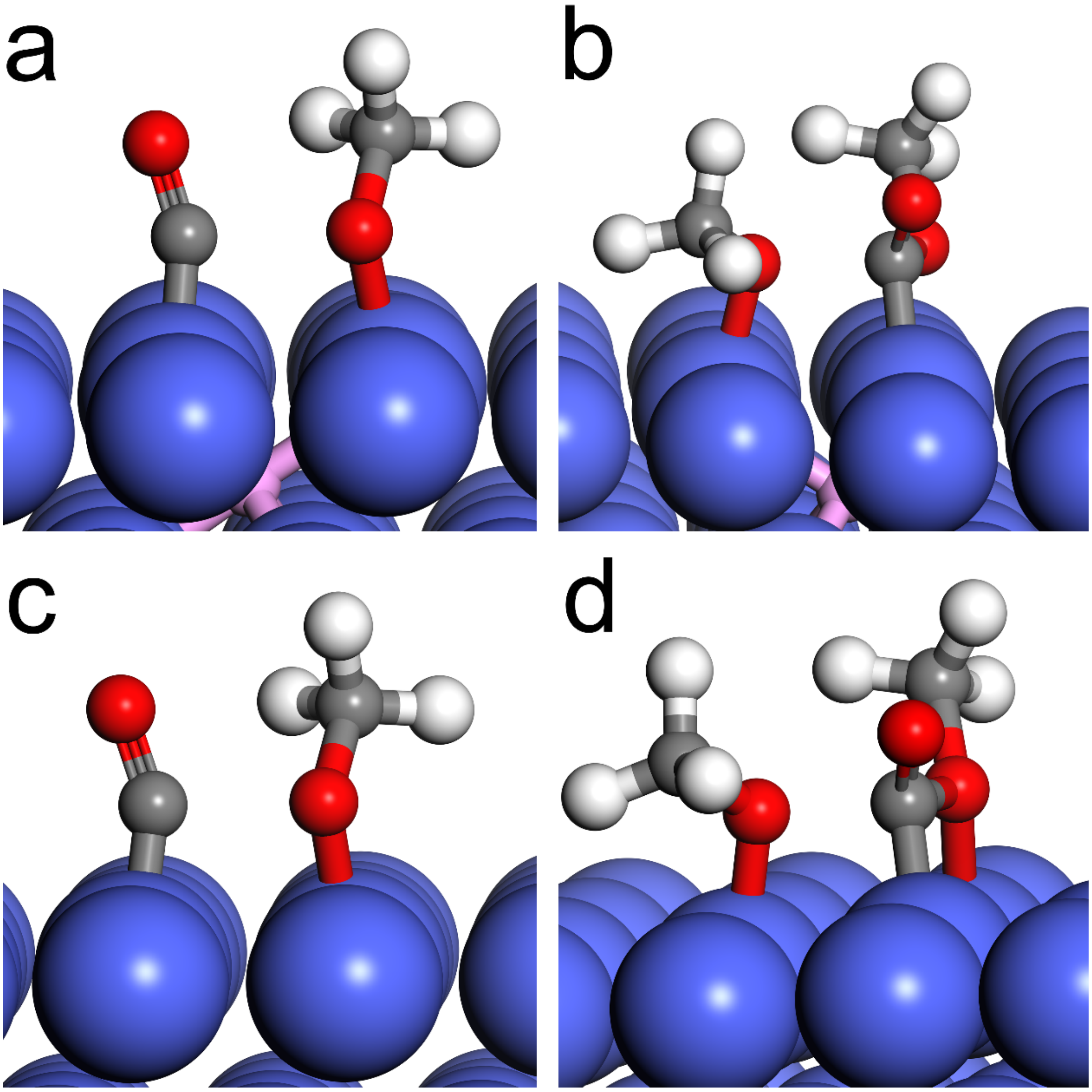

Supplementary Figure 4. Geometries of transition states of OC-OCH_3 on (a) Pd-B and (c) Pd, and transition states of CH_3O-C(O)OCH_3 on (b) Pd-B, and (d) Pd in DMC formation reactions. Red, grey, white, pink, and blue balls represent oxygen, carbon, hydrogen, boron, and palladium, respectively.

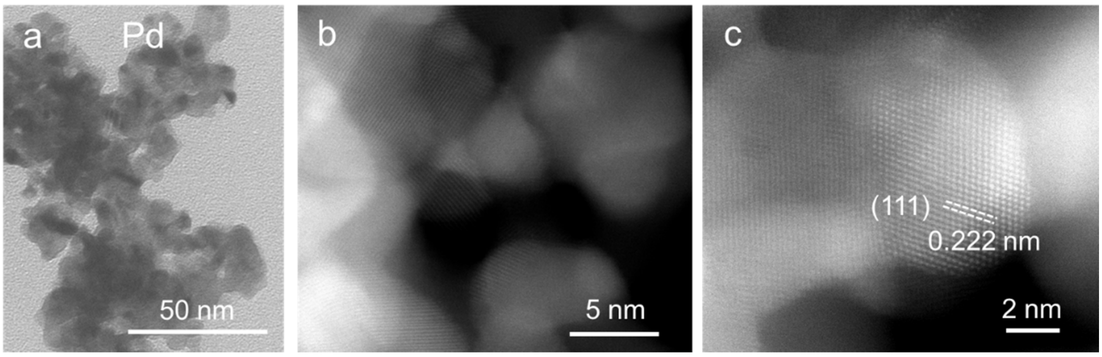

Supplementary Figure 5. (a) TEM, (b) HAADF, and (c) HAADF-STEM images of pure Pd nanomaterial, showing the similar morphology and size with Pd-B sample; while smaller lattice spacing of (111) facet compared to that of Pd-B.

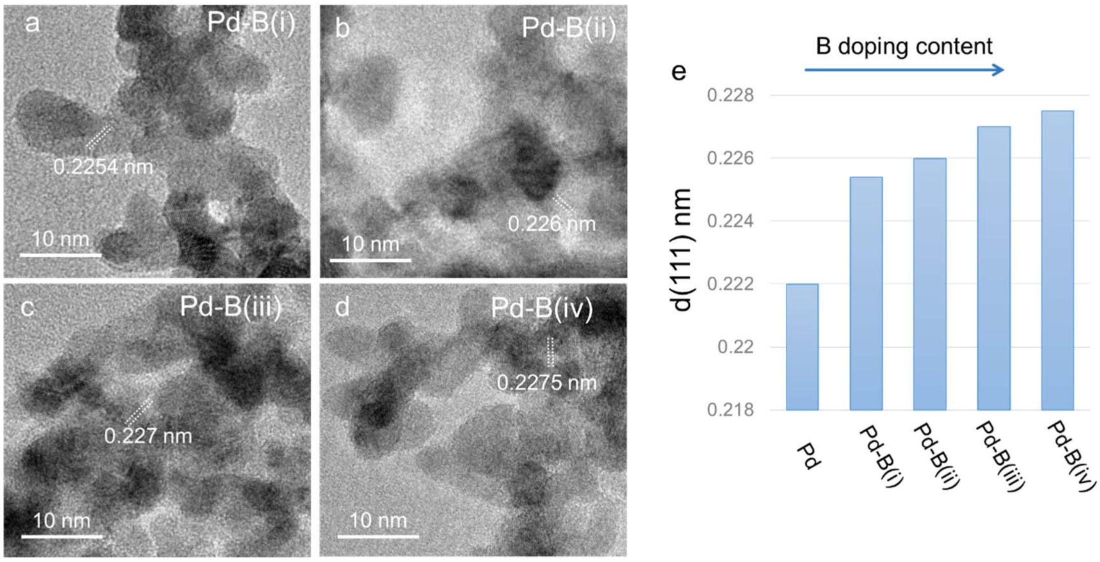

Supplementary Figure 6. (a-d) HRTEM images of Pd-B nanomaterials. (e) The lattice spacing of (111) facet versus Pd-based sample, showing the lattice fringes expanded with B doping content increased.

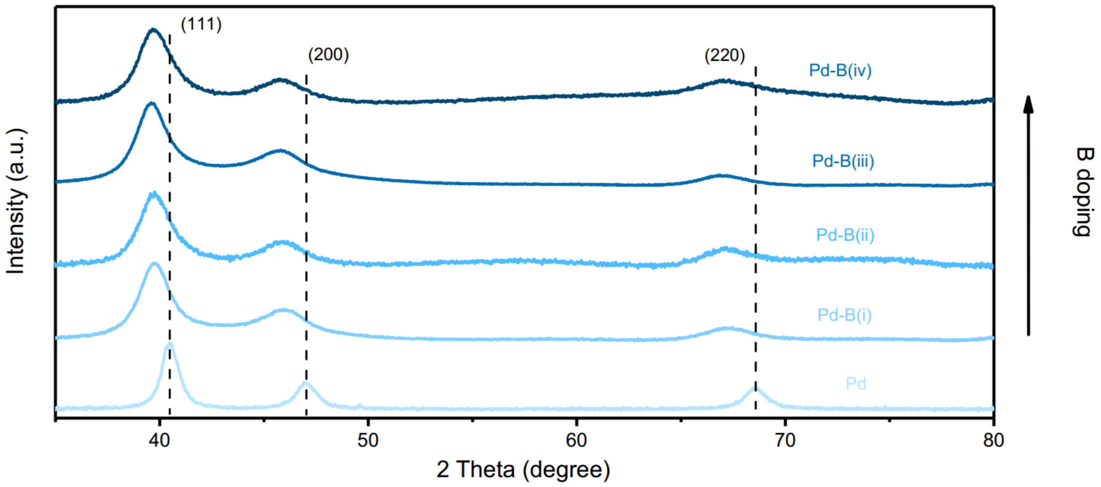

Supplementary Figure 7. Powder XRD spectra of Pd-B and Pd samples, showing the peaks shift more with B doping content increased.

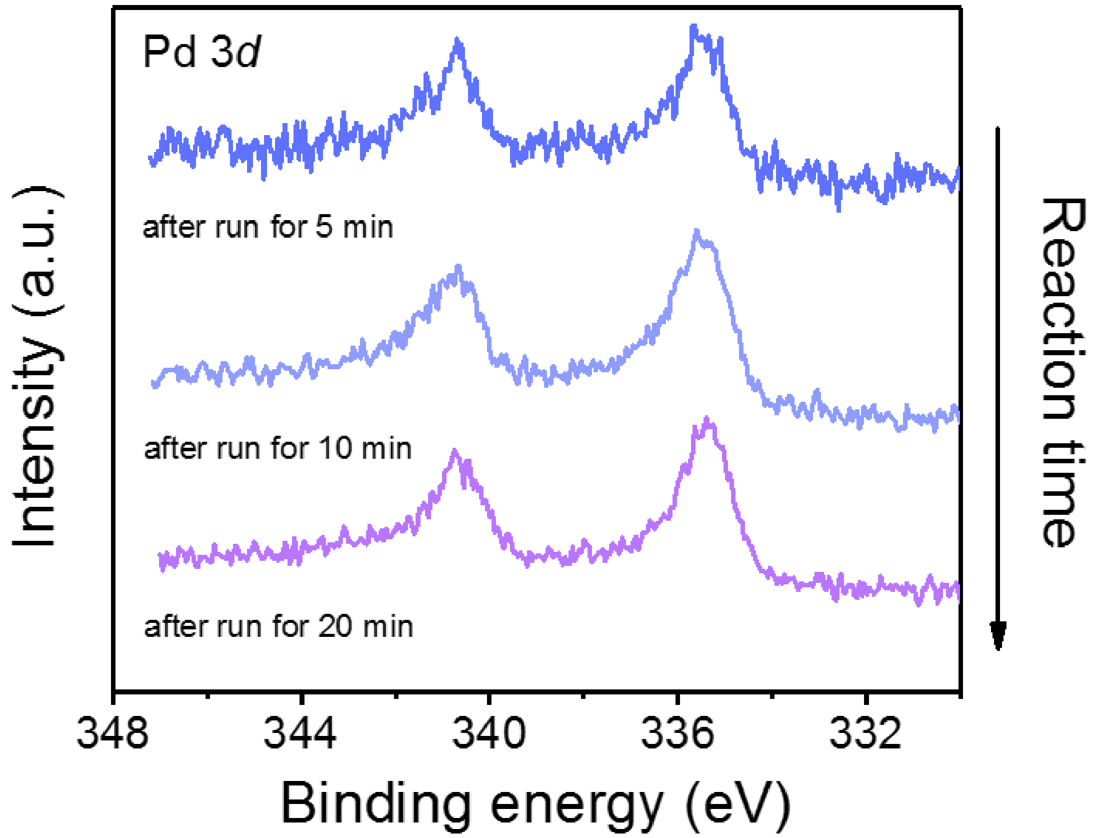

Supplementary Figure 8. XPS spectra of the Pd-B sample with time evolution of electroxidation reaction, demonstrating the Pd valence state is stable.

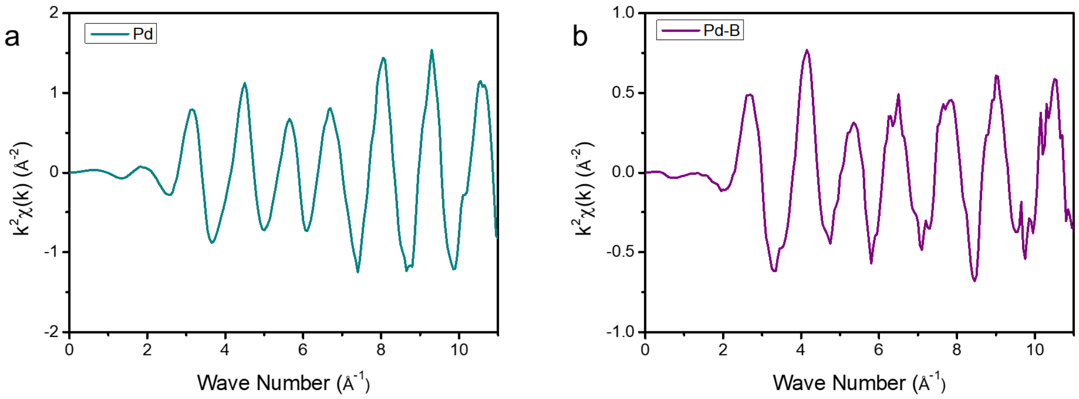

Supplementary Figure 9. Pd K-edge XAS oscillation function. $k^2\chi(k)$ of (a) Pd and (b) Pd-B were measured during the DMC electroproduction at 1.4 V vs. Ag/AgCl.

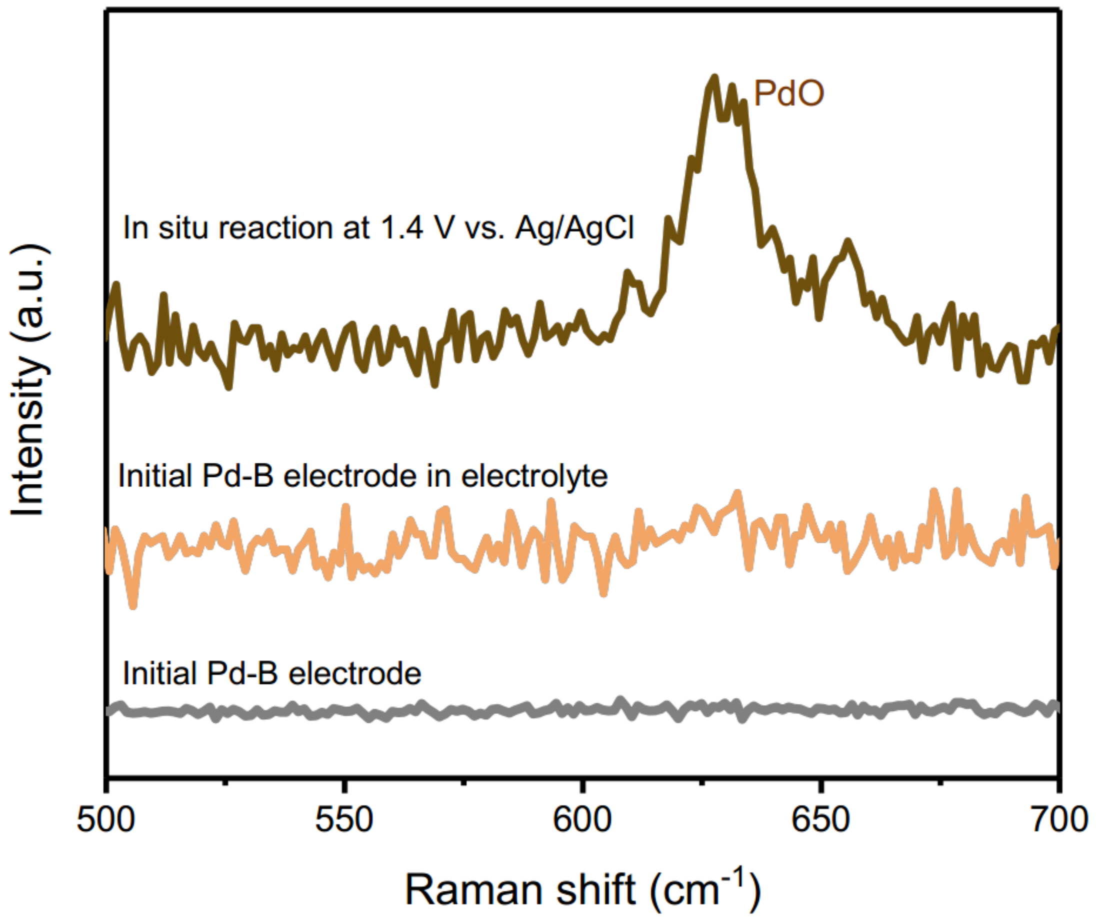

Supplementary Figure 10. Raman spectroscopic measurements on Pd-B electrode. We tested the initial Pd-B electrode (gray curve), the initial electrode in the electrolyte of 0.1 M $NaClO_4$/methanol (orange curve), and the electrode in the electrolyte run at the potential of 1.4 V vs. Ag/AgCl without CO gas bubbling (brown curve). The results show that Pd^0 could be oxidized to Pd^{2+} without CO in the electrolyte with applied potential, as evidenced by observed peak (at ca. 630 cm^{-1})[20], in agreement with the CV result shown in Fig. 4a left.

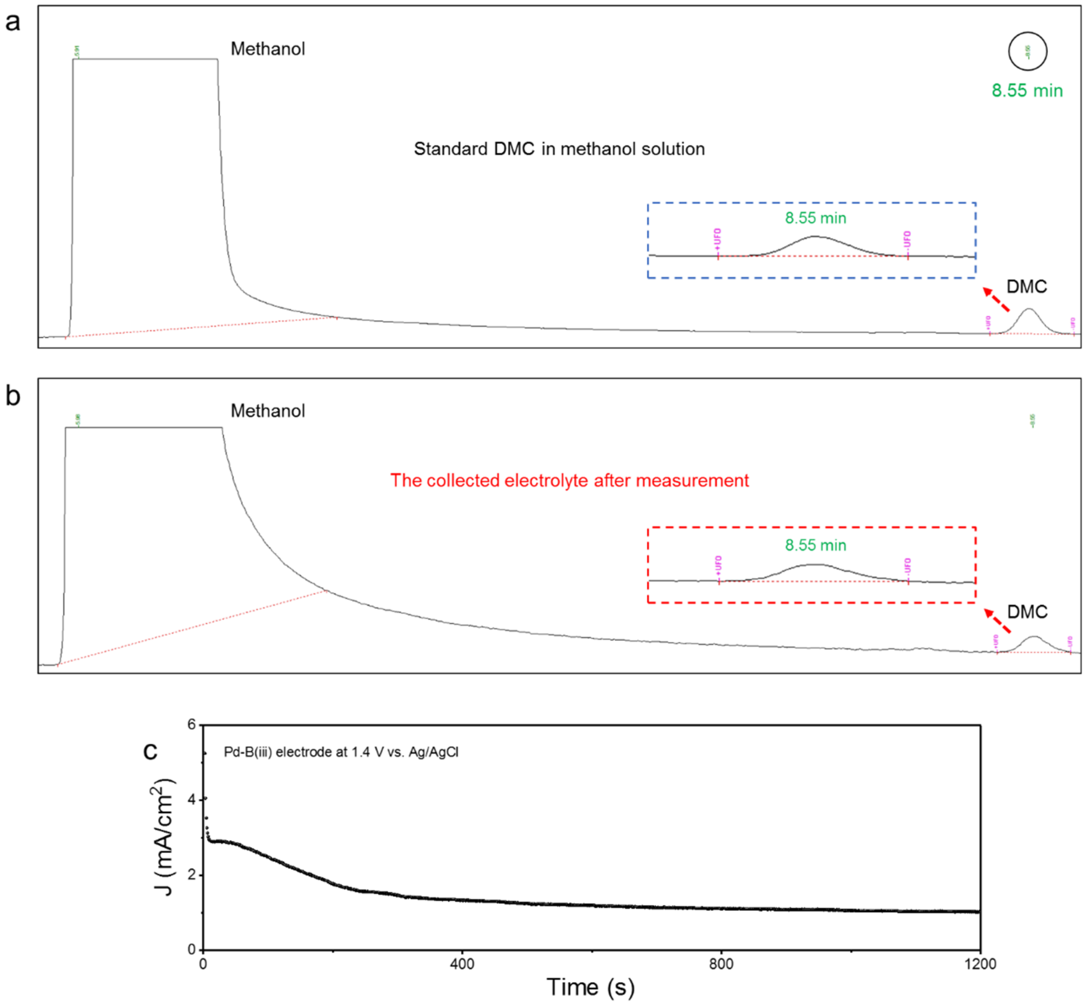

Supplementary Figure 11. GC-FID traces. (a) The commercial DMC in methanol solution. (b) The collected electrolyte after the test using the Pd-B(iii) catalyst under 1.4 V vs Ag/AgCl for 20 min (c).

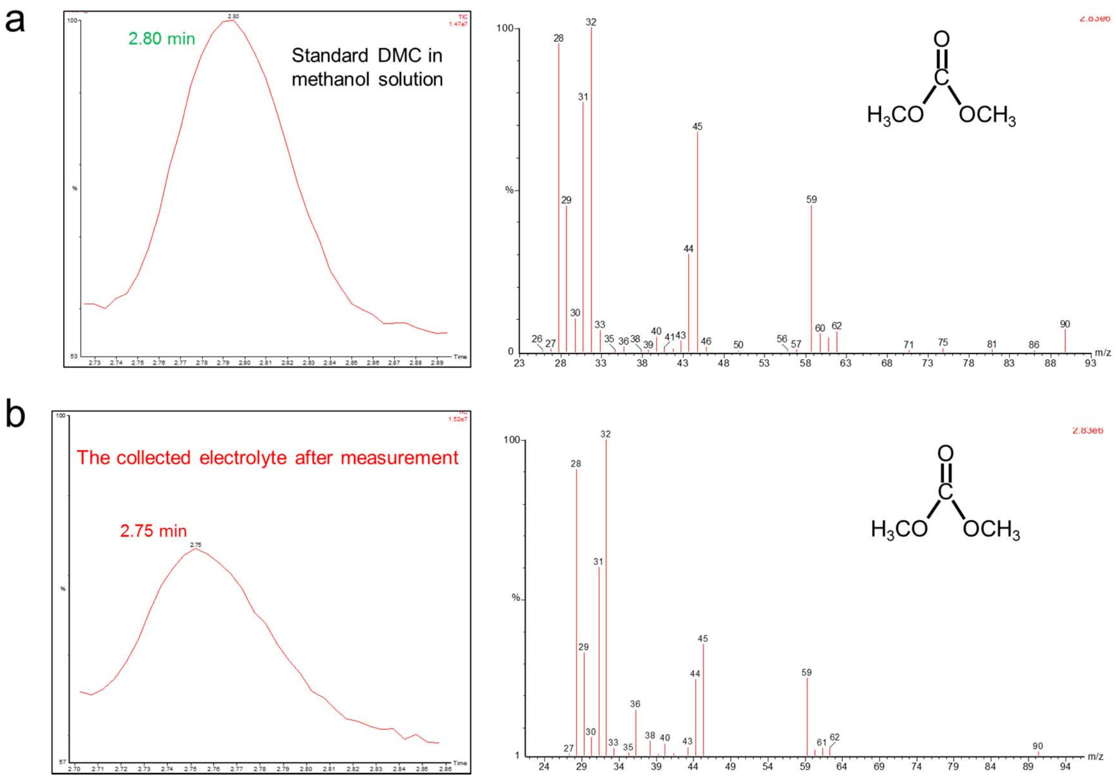

Supplementary Figure 12. GC-MS spectra of (a) the commercial DMC in methanol solution and (b) the collected electrolyte after test, respectively.

Supplementary Table 1. The total costs of electrosynthesized DMC with different DMC Faradaic efficiencies.

DMC Faradaic efficiency (%)	The total costs of DMC (US$/ton)
10	1925.30
20	1185.83
30	939.34
40	816.10
50	742.15
60	692.85
70	657.64
80	631.23
90	610.69
100	594.25

Supplementary Table 2. Free energy values in Fig. 1d. All energies are in eV.

U=1 V vs. SHE	Pd	Pd-B
$CO(g)+2CH_3OH(l)$	0.00	0.00
$CO^*+2CH_3OH(l)$	-1.85	-1.16
$CO^*+CH_3O^*+H^++e^-+CH_3OH(l)$	-2.58	-1.25
$CO^* + 2CH_3O^*+2H^++2e^-$	-3.32	-1.34
$OC\text{-}OCH_3^*+CH_3O^*+2H^++2e^-$	-1.82	-0.77
$OCOCH_3^*+CH_3O^* + 2H^++2e^-$	-2.38	-1.54
$CH_3OC(O)\text{-}OCH_3^*+2H^+ + 2e^-$	-1.56	-1.26
$DMC(l)+2H^++2e^-$	-2.20	-2.20

Supplementary Table 3. Stabilities of boron doped on different site. All the energies are in eV and with respect to the energy of the most stable structure.

B site	stability
subsurface fcc	0.00
subsurface hcp	1.16
surface hcp	1.40
surface fcc	1.44

Supplementary Table 4. Bader charge of B and the connected three surface and subsurface Pd.

Atom	Bader charge
B	0.396
sub-Pd1	-0.010
sub-Pd2	-0.010
sub-Pd3	0.010
surf_Pd1	-0.060
surf_Pd2	-0.060
surf_Pd3	-0.060

Supplementary Table 5. The adsorption energies of CO (E(CO)) and the free energy change of CH_3OH dissociation ($\Delta G(CH_3OH)$) on Pd(111) with boron number from 0 to 10. All energies are in eV.

	$\Delta G(CH_3OH)$	E(CO)
Pd(111)	-0.73	-1.85
Pd(111)-1B	-0.09	-1.15
Pd(111)-2B	-0.09	-1.15
Pd(111)-3B	-0.04	-1.17
Pd(111)-4B	-0.02	-1.04
Pd(111)-5B	-0.13	-0.89
Pd(111)-6B	-0.11	-0.90
Pd(111)-7B	-0.07	-0.82
Pd(111)-8B	-0.13	-0.77
Pd(111)-9B	-0.19	-0.69
Pd(111)-10B	-0.09	-0.65

Supplementary Table 6. XANES linear combination fitting of pure Pd and Pd-B.

	Average oxidation state of Pd
Pure Pd (before reaction)	0.494
Pure Pd (in-situ at 1.4 V)	0.588
Pd-B (before reaction)	0.604
Pd-B (in-situ at 1.4 V)	0.678

Supplementary Table 7. EXAFS fitting for Pd-Pd and Pd-B coordination number and interatomic distance comparison between pure Pd and Pd-B.

	Pd in situ (1.4 V)	Pd-B in situ (1.4 V)
Pd-Pd CN	10.4	9.65
Pd-Pd R (Å)	2.7466	2.7764
Pd-Pd σ^2	0.0060	0.0102
Pd-Pd ΔE_0	0.89	-6.86
Pd-B CN	--	1.98
Pd-B R (Å)	--	2.1154
Pd-B σ^2	--	0.0102
Pd-B ΔE_0	--	-0.16

Supplementary Table 8. Faradaic efficiencies of DMC at different applied potentials (V vs. Ag/AgCl) using different samples.

	Pd	Pd-B(i)	Pd-B(ii)	Pd-B(iii)	Pd-B(iv)
1.0	15.0 ± 2%	/	39.8 ± 3%	51.0 ± 4%	/
1.2	/			54.5 ± 4%	37.5 ± 2%
1.3	/	28.2 ± 3%	/	56.4 ± 5%	52.8 ± 4%
1.4	24.8 ± 2%	/	50.4 ± 4%	83.3 ± 5%	75.2 ± 5%
1.5	29.7 ± 3%	42.7 ± 4%	73.2 ± 4%	36.0 ± 3%	52.0 ± 5%
1.6	20.6 ± 2%	33.1 ± 5%	46.8 ± 4%	/	21.6 ± 3%

Supplementary Table 9. Comparison of DMC Faradaic efficiency.

Catalyst	FE (%)	Applied potential (V)	Reference
$PdCl_2$/vapor grown carbon fiber	60	1.8 V vs. Ag/AgCl	Ref[21]
Au/carbon	35	1.5 V vs. Ag/AgCl	Ref[22]
Pd/vapor grown carbon fiber	67	1.8 V vs. Ag/AgCl	Ref[23]
$HAuCl_4$/active carbon	5	1.5 V vs. Ag/AgCl	Ref[24]
Copper carbonyl species	6	0.1 V vs. SCE	Ref[25]
Pd-B(iii)	83	1.4 V vs Ag/AgCl	This work

Supplementary Table 10. The B/Pd atomic content in Pd-based catalysts.

Catalyst	Pd	Pd-B(i)	Pd-B(ii)	Pd-B(iii)	Pd-B(iv)
B/Pd content	0	0.1	0.16	0.19	0.22

Supplementary Reference

1. He, C. & You, F.Q. Shale Gas Processing Integrated with Ethylene Production: Novel Process Designs, Exergy Analysis, and Techno-Economic Analysis. *Ind. Eng. Chem. Res.* **53**, 11442-11459 (2014).
2. Weekes, D.M., Salvatore, D.A., Reyes, A., Huang, A. & Berlinguette, C.P. Electrolytic CO_2 Reduction in a Flow Cell. *Acc. Chem. Res.* **51**, 910-918 (2018).
3. Bushuyev, O.S. et al. What Should We Make with CO_2 and How Can We Make It? *Joule* **2**, 825-832 (2018).
4. Kresse, G. & Furthmüller, J. Efficient iterative schemes for ab initio total-energy calculations using a plane-wave basis set. *Phys. Rev. B* **54**, 11169-11186 (1996).
5. Kresse, G. & Furthmuller, J. Efficiency of ab-initio total energy calculations for metals and semiconductors using a plane-wave basis set. *Comp. Mater. Sci.* **6**, 15-50 (1996).
6. Kresse, G. & Hafner, J. Ab-Initio Molecular-Dynamics Simulation of the Liquid-Metal Amorphous-Semiconductor Transition in Germanium. *Phys. Rev. B* **49**, 14251-14269 (1994).
7. Kresse, G. & Hafner, J. Ab initio molecular dynamics for liquid metals. *Phys. Rev. B* **47**, 558-561 (1993).
8. Perdew, J.P., Burke, K. & Ernzerhof, M. Generalized Gradient Approximation Made Simple. *Phys. Rev. Lett.* **77**, 3865-3868 (1996).
9. Kresse, G. & Joubert, D. From ultrasoft pseudopotentials to the projector augmented-wave method. *Phys. Rev. B* **59**, 1758-1775 (1999).
10. Blöchl, P.E. Projector augmented-wave method. *Phys. Rev. B* **50**, 17953-17979 (1994).
11. Grimme, S., Antony, J., Ehrlich, S. & Krieg, H. A consistent and accurate ab initio parametrization of density functional dispersion correction (DFT-D) for the 94 elements H-Pu. *J. Chem. Phys.* **132**, 154104 (2010).
12. Michaelides, A. et al. Identification of general linear relationships between activation energies and enthalpy changes for dissociation reactions at surfaces. *J. Am. Chem. Soc.* **125**, 3704-3705 (2003).
13. Liu, Z.-P. & Hu, P. General rules for predicting where a catalytic reaction should occur on metal surfaces: a density functional theory study of C− H and C− O bond breaking/making on flat, stepped, and kinked metal surfaces. *J. Am. Chem. Soc.* **125**, 1958-1967 (2003).
14. Alavi, A., Hu, P., Deutsch, T., Silvestrelli, P.L. & Hutter, J. CO oxidation on Pt (111): An ab initio density functional theory study. *Phys. Rev. Lett.* **80**, 3650 (1998).
15. Mathew, K., Sundararaman, R., Letchworth-Weaver, K., Arias, T.A. & Hennig, R.G. Implicit solvation model for density-functional study of nanocrystal surfaces and reaction pathways. *J. Chem. Phys.* **140**, 084106 (2014).
16. Shirke, R.M., Chaudhari, A., More, N.M. & Patil, P.B. Temperature dependent dielectric

relaxation study of ethyl acetate — Alcohol mixtures using time domain technique. *J. Mol. Liq.* **94**, 27-36 (2001).

17. NIST Chemistry WebBook, NIST Standard Reference Database Number 69. (National Institute of Standards and Technology, 2005).
18. Zhou, Y., Wu, J. & Lemmon, E.W. Thermodynamic Properties of Dimethyl Carbonate. *J. Phys. Chem. Ref. Data* **40**, 043106 (2011).
19. Fiorani, G., Perosa, A. & Selva, M. Dimethyl carbonate: a versatile reagent for a sustainable valorization of renewables. *Green Chem.* **20**, 288-322 (2018).
20. Demoulin, O. et al. Operando resonance Raman spectroscopic characterisation of the oxidation state of palladium in Pd/γ-Al_2O_3 catalysts during the combustion of methane. *Phys. Chem. Chem. Phys.* **5**, 4394-4401 (2003).
21. Yamanaka, I., Funakawa, A. & Otsuka, K. Selective Carbonylation of Methanol to Dimethyl Carbonate by Gas–Liquid–Solid-Phase Boundary Electrolysis. *Chem. Lett.* **31**, 448-449 (2002).
22. Funakawa, A., Yamanaka, I. & Otsuka, K. Active control of methanol carbonylation selectivity over Au/carbon anode by electrochemical potential. *J. Phys. Chem. B* **109**, 9140-9147 (2005).
23. Yamanaka, I., Funakawa, A. & Otsuka, K. Electrocatalytic synthesis of DMC over the Pd/VGCF membrane anode by gas–liquid–solid phase-boundary electrolysis. *J. Catal.* **221**, 110-118 (2004).
24. Funakawa, A., Yamanaka, I., Takenaka, S. & Otsuka, K. Selectivity control of carbonylation of methanol to dimethyl oxalate and dimethyl carbonate over gold anode by electrochemical potential. *J. Am. Chem. Soc.* **126**, 5346-5347 (2004).
25. Davies, B.J. et al. Electrochemically generated copper carbonyl for selective dimethyl carbonate synthesis. *ACS Catal.* **9**, 859-866 (2018).
